# Comment on Oral White Lesions Associated with Chewing Khat

**DOI:** 10.1186/1617-9625-3-1-5

**Published:** 2005-12-15

**Authors:** Ali Aiman

**Affiliations:** 1 P O Box 60710 Dammam 31555 Saudi Arabia

## 

Dear Editor,

I have read the nice paper published in your estimable journal of Tobacco Induced Diseases Vol. 2, No. 3: 145-150 (2004) about "Oral White Lesions Associated with Chewing Khat". This was a clinical descriptive study done by Gorsky et al. on 47 Yemeni Israeli who had chewed khat more than 3 years and 55 Yemeni non-chewers as group control.

I kindly wanted to express my thoughts about some points mentioned in the paper including:

1. The phrase [chewing khat] mentioned in the paper is a misnomer and should be replaced by "takhzeen al-qat" as chewing does not infer the exact meaning of what Yemeni people used to do. They used to do "takhzeen" which means in Arabic chewing and storing of qat for several hours. Therefore, the Arabic word takhzeen is used to properly describe this habit1. And the word qat with letter "q" is more commonly used than khat, particularly in the recent studies [1-3].

2. The authors addressed people who chewed qat for more than 3 years (neglecting the frequency) as chronic chewers, in fact chronocity is dependent on two main factors; the duration (time in years), and frequency of chewing per week. For instance, many persons may use qat for more than 10 years in low frequency (once per month), yet, not considered as chronic users. On the other hand, others may practise it daily for only 2 years and considered as chronic. A recent study strongly correlated the effect of this habit with the frequency

3. The authors mentioned in their study a very exaggerated percentages of white lesions in both control (16%) and chewer (83%) groups. Whereas the highest international reported incidence4 of oral white lesions among the normal population is not more than 11.6% and among qat chewers1 (n = 1528) is 22.3%. Furthermore, the authors did not mention any description for the white lesions they found in the control group. What types of white lesions were found?

4. The authors addressed 14.6% of the diagnosed white lesions as non-homogenous, at the same time they said "No white lesion was felt to be clinically suspicious for malignant or premalignant changes", on the other hand, non-homogenous white lesions are considered in the international reports as aggressive lesions [5-7]. Moreover, the occurrence of such lesions is in contrary with most of other studies which reported that takhzeen al-qat may only cause homogenous or benign changes in the oral and esophagus mucosa [1,8-10].

5. Finally, all recent references cited in this paper are not related to qat habit but to other habits in other regions of the world. While authors did not quote several studies done in the last 10 years on this habit in the Middle East, which are available through the Medline[1-3,11,12].

Looking forward to hearing from you

Yours sincerely

Ali A. A.

## Competing interests

The authors declare that they have no competing interests.
